# Preoperative Mural Thrombus Volume Ratio Predicts Aneurysm Enlargement in Type 2 Endoleak after Endovascular Aortic Repair

**DOI:** 10.3400/avd.oa.26-00001

**Published:** 2026-04-01

**Authors:** Yuki Oga, Tomoaki Kudo, Yasuka Nakanishi, Mitsuru Yuzaki, Tomohiko Sakamoto, Yusuke Ki, Toru Kuratani

**Affiliations:** Department of Cardiovascular Surgery, Osaka International Medical & Science Center, Osaka, Osaka, Japan

**Keywords:** endovascular aortic repair, type 2 endoleak, lumbar artery, aneurysm enlargement

## Abstract

**Objectives:**

This study aimed to identify anatomical predictors of aneurysm enlargement in patients with lumbar artery (LA)–related type 2 endoleak (T2EL) after endovascular aortic repair (EVAR).

**Methods:**

This retrospective observational cohort study included 59 patients with infrarenal abdominal aortic aneurysms who developed T2EL caused exclusively by LAs after EVAR between January 2019 and July 2024. Computed tomography–based anatomical parameters, including mural thrombus volume ratio, calcified volume ratio, aneurysm diameter, and LA anatomy, were analyzed.

**Results:**

During a median follow-up of 33 months, aneurysm enlargement occurred in 9 patients (15.3%). Patients with aneurysm enlargement had a significantly lower mural thrombus volume ratio (*p* <0.001). Female sex (hazard ratio [HR], 0.23; 95% confidence interval [CI], 0.06–0.84; *p* = 0.026) and mural thrombus volume ratio (HR, 0.88 per 1% increase; 95% CI, 0.80–0.96; *p* = 0.003) were significantly associated with aneurysm enlargement, whereas LA anatomy was not. Receiver operating characteristic analysis identified an optimal cutoff value of 29.2% (area under the curve, 0.82).

**Conclusions:**

A low preoperative mural thrombus volume ratio was associated with aneurysm enlargement in patients with LA–related T2EL after EVAR. This parameter may serve as a practical imaging biomarker to support selective consideration of preemptive embolization.

## Introduction

Endoleak is a well-recognized complication following endovascular aortic repair (EVAR) and remains one of the leading causes of postoperative reintervention.^1–3)^ Among the various endoleak subtypes, type 2 endoleak (T2EL) is the most common and results from retrograde flow through aortic branch vessels, such as the inferior mesenteric artery (IMA) and lumbar arteries (LAs).^3,4)^ Although many cases of T2EL resolve spontaneously, persistent endoleak can promote aneurysm enlargement and is associated with an increased risk of rupture, aneurysm-related mortality, and secondary interventions.^1,2,5)^

Preemptive embolization of aortic side branches has been introduced as a strategy to reduce the incidence of T2EL and subsequent aneurysm enlargement.^6,7)^ While embolization of the IMA has demonstrated high technical success, embolization of the LAs remains technically demanding because of anatomical factors, including large aortic diameters and difficulty in catheterization.^4,8)^ In addition, LA embolization is associated with longer operative times, increased radiation exposure, and greater use of contrast agents. As a result, routine embolization of all LAs is not feasible in many clinical settings, and the indications for LA embolization remain controversial.^9,10)^

Given these limitations, complete prevention of T2EL is unrealistic in routine clinical practice. Instead, the clinically relevant challenge is identifying patients in whom T2EL leads to aneurysm enlargement, as this subgroup carries the greatest risk of adverse outcomes and may benefit from targeted intervention.^1,2)^ Previous studies have primarily focused on branch vessel–related factors, such as the number and diameter of patent aortic side branches or hemodynamic parameters, to predict aneurysm enlargement.^4,11,12)^ However, data specifically addressing T2EL caused exclusively by LAs are limited, and the role of aneurysm wall–related factors, such as mural thrombus, has not been fully elucidated.^13,14)^

The present study aimed to identify anatomical predictors of aneurysm enlargement in patients with T2EL caused solely by LAs after EVAR. In particular, we focused on the preoperative mural thrombus volume ratio assessed by computed tomography (CT) and evaluated its potential role as a practical imaging biomarker for risk stratification and selective consideration of preemptive LA embolization.

## Materials and Methods

### Study population

**Fig. 1:**
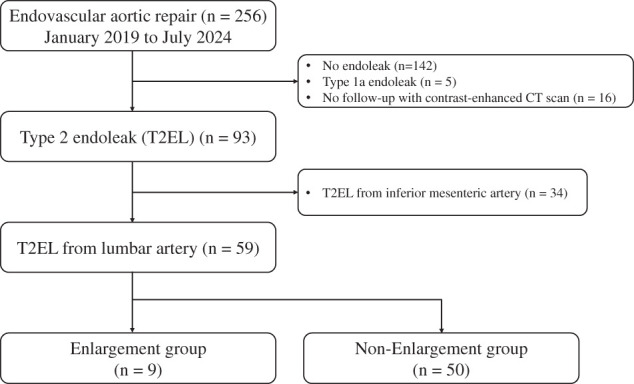
Patient flowchart. Flow diagram illustrating patient selection for the study. Between January 2019 and July 2024, 256 patients underwent EVAR. After exclusion of patients without T2EL, with type 1a endoleak, or without postoperative contrast-enhanced CT follow-up, 93 patients with T2EL were identified. Among these, 34 patients with inferior mesenteric artery–related T2EL were excluded, leaving 59 patients with lumbar artery–related T2EL for analysis. These patients were classified into the aneurysm enlargement group (n = 9) and the non-enlargement group (n = 50). EVAR: endovascular aneurysm repair; T2EL: type 2 endoleak; CT: computed tomography

This retrospective observational cohort study included 256 consecutive patients with infrarenal abdominal aortic aneurysms who underwent EVAR between January 2019 and July 2024. Of these, patients without any endoleaks (n = 142), with type 1a endoleak (n = 5), or without postoperative contrast-enhanced CT follow-up (n = 16) were excluded. Consequently, 93 patients were diagnosed with T2EL. Among these 93 patients, 34 with T2EL originating from the IMA were excluded, leaving 59 patients with T2EL caused exclusively by LAs for the final analysis (**Fig. 1**).

EVAR was performed according to contemporary clinical practice guidelines and our institutional decision-making. Although aneurysm diameter ≥50 mm was the principal indication for repair, additional indications included rapid aneurysm growth (>5 mm within 6 months), symptomatic aneurysm, saccular morphology, and anatomically high-risk features considered prone to rupture. In selected cases, lower diameter thresholds were applied based on patient-specific risk factors and overall clinical judgment, particularly in female patients or in cases with unfavorable anatomical characteristics.

### Modality and measurements

Calcified volume ratio and mural thrombus volume ratio were calculated using CT imaging (**Supplementary Fig. 1**). A 3-dimensional reconstruction of the entire abdominal aorta was performed using contrast-enhanced CT, capturing the region from just below the lower renal artery to the distal end of the terminal aorta. Subsequently, non-contrast CT images were used to extract the corresponding regions, followed by nonrigid registration. The total calcified volume and thrombus volume were derived by subtracting the area delineated by contrast-enhanced CT from that of the non-contrast CT. The Agatston score was employed to identify calcified regions (threshold >130 Hounsfield units), and this value was subtracted from the total non-contrast volume to isolate thrombus volume. The calcified volume ratio and mural thrombus volume ratio were expressed as percentages relative to the total abdominal aortic volume.

All anatomical data were retrospectively collected for each patient based on contrast-enhanced CT imaging. Image acquisition and initial anatomical assessment were performed collaboratively by radiology technologists and cardiovascular surgeons. Subsequently, detailed volumetric analyses, including mural thrombus and calcified volume measurements, were conducted by a single experienced investigator who was blinded to clinical outcomes.

LA diameters were measured on arterial-phase contrast-enhanced CT angiography. For each patent LA, the diameter was assessed at its origin from the abdominal aorta on axial images, with reference to multiplanar reconstructions when necessary. Measurements were performed using a standardized protocol applied uniformly across all patients, and the maximum LA diameter was used for analysis.

### Follow-up protocol

Endoleak was assessed using contrast-enhanced CT scans. The first postoperative contrast-enhanced CT scan was performed 7 days after EVAR to evaluate early endoleak status. Subsequent CT examinations were obtained at 1, 6, and 12 months postoperatively and annually thereafter.

Measurements of aneurysm diameter were performed at each follow-up visit. Aneurysm enlargement was defined as an increase of ≥5 mm in the maximum aneurysm diameter compared with the postoperative baseline measurement, whereas aneurysm shrinkage was defined as a decrease of ≥5 mm.

For time-to-event analysis, the time to aneurysm enlargement was defined as the interval between the date of EVAR and the date of the CT scan at which aneurysm enlargement was first detected. Patients without aneurysm enlargement were censored at the date of the last available CT follow-up.

### Statistical analysis

Continuous data are expressed as the mean ± SD or as the median with interquartile range (IQR), depending on the normality of data distribution, which was assessed using the Shapiro–Wilk test. Comparisons were performed using the Mann–Whitney U test. Categorical variables are presented as counts and percentages and were compared using the chi-squared test or Fisher’s exact test.

Univariate Cox proportional hazards regression analysis was performed to examine the risk factors for aneurysm enlargement associated with T2EL caused solely by LAs throughout the follow-up period.

To test the discriminatory value of independent predictors of aneurysm enlargement, we used the area under the curve (AUC) method. We also used receiver operating characteristic (ROC) analysis to investigate the cutoff value for each factor that maximized the AUC for aneurysm enlargement.

Curves for aneurysm enlargement were estimated using the Kaplan–Meier product limit method and compared using log-rank tests. Estimates are provided with 95% confidence intervals (CIs).

All *p* values are 2-sided, and statistical significance was set at *p* <0.05. Statistical analyses were performed using JMP statistical software, version 17.0.0 for MacOS X (SAS Institute, Cary, NC, USA).

## Results

### Baseline characteristics

The median follow-up duration was 33 months (IQR, 14–51 months). The baseline characteristics of the 59 patients included in this study are summarized in **Table 1**. During the follow-up period, aneurysm enlargement was observed in 9 patients (15.3%), whereas the remaining 50 patients (84.7%) showed no evidence of aneurysm enlargement on postoperative contrast-enhanced CT.

**Table 1:** Baseline characteristics and computed tomography measurement

	Total (n = 59)	Enlargement (n = 9)	Non-enlargement (n = 50)	*p* Value
Baseline characteristics				
Age (years)	78.3 ± 7.5	79.4 ± 6.8	78.1 ± 7.7	0.61
Male, n (%)	44 (74.6)	4 (44.4)	40 (80.0)	0.038
Preoperative complications				
Smoking history, n (%)	43 (72.9)	4 (44.4)	39 (78.0)	0.052
Hypertension, n (%)	43 (72.9)	6 (66.7)	37 (74.0)	0.69
Dyslipidemia, n (%)	33 (55.9)	5 (55.6)	28 (56.0)	1.00
Diabetes mellitus, n (%)	3 (5.1)	0	3 (6.0)	1.00
Coronary artery disease, n (%)	19 (32.2)	1 (11.1)	18 (36.0)	0.25
Cerebrovascular disease, n (%)	11 (18.6)	2 (22.2)	9 (18.0)	0.67
CKD stage ≥4, n (%)	25 (42.4)	4 (44.4)	21 (42.0)	1.00
Type of stent grafts				
Gore Excluder, n (%)	51 (86.4)	9 (100)	42 (84.0)	0.34
Medtronic Endurant, n (%)	8 (13.6)	0 (0.0)	8 (16.0)	0
CT measurement				
Maximal aneurysm diameter (mm)	47.0 (41.0–51.0)	45.0 (40.0–54.0)	47.0 (41.5–50.0)	0.94
Abdominal aorta				
Total volume (mL)	127 (105–169)	127 (89–186)	126 (108–167)	0.71
Mural thrombus volume (mL)	41.6 (24.1–65.5)	19.0 (11.0–27.0)	44.4 (28.6–70.9)	0.002
Mural thrombus volume ratio (%)	32.0 ± 16.6	14.0 ± 7.9	35.3 ± 15.7	<0.001
Calcified volume (mL)	5.4 (2.9–10.3)	5.6 (2.8–9.9)	5.3 (3.1–10.3)	0.99
Calcified volume ratio (%)	3.8 (1.9–6.9)	5.1 (1.4–6.6)	3.7 (2.2–7.0)	0.98
Patent LAs				
Number (n)	4.0 (3.0–5.0)	4.0 (4.0–4.0)	4.0 (3.0–5.0)	0.66
Maximum diameter (mm)	1.7 (1.5–2.1)	1.6 (1.3–2.1)	1.8 (1.5–2.1)	0.71

Data are presented as mean ± standard deviation and median (IQR)

CKD: chronic kidney disease; CT: computed tomography; LA: lumbar artery; IQR: interquartile range

The mean age of the study population was 78.3 ± 7.5 years, and 44 patients (74.6%) were male. The majority of patients were treated with the Excluder stent graft (W. L. Gore & Associates, Flagstaff, AZ, USA) (n = 51, 86.4%), while the Endurant stent graft (Medtronic, Santa Rosa, CA, USA) was used in 8 patients (13.6%). Comparison between the aneurysm enlargement and non-enlargement groups revealed a significantly higher proportion of female patients in the enlargement group (*p* = 0.04). No other baseline demographic or clinical characteristics differed significantly between the 2 groups.

### CT findings and anatomical characteristics

CT–based anatomical measurements are summarized in **Table 1**. The median maximal aneurysm diameter did not differ significantly between the aneurysm enlargement and non-enlargement groups (45.0 vs. 47.0 mm, *p* = 0.94). Similarly, total abdominal aortic volume, calcified volume, and calcified volume ratio were comparable between the 2 groups.

In contrast, mural thrombus volume and mural thrombus volume ratio were significantly lower in patients who developed aneurysm enlargement. Median mural thrombus volume was 19.0 mL (IQR, 11.0–27.0 mL) in the enlargement group compared with 44.4 mL (IQR, 28.6–70.9 mL) in the non-enlargement group (*p* = 0.002). Likewise, the mean mural thrombus volume ratio was significantly lower in the enlargement group than in the non-enlargement group (14.0 ± 7.9% vs. 35.3 ± 15.7%, *p* <0.001).

With respect to LA anatomy, neither the number of patent LAs nor their maximum diameter differed significantly between the 2 groups (both *p* >0.60).

### Risk factors for aneurysm enlargement

Univariate analysis for predictors of aneurysm enlargement is shown in **Table 2**. In univariate analysis, female sex (hazard ratio [HR], 0.23; 95% CI, 0.06–0.84; *p* = 0.026) and mural thrombus volume ratio (HR, 0.88 per 1% increase; 95% CI, 0.80–0.96; *p* = 0.003) were significantly associated with aneurysm enlargement. Other variables, including age, stent graft type, maximal aneurysm diameter, calcified volume ratio, number of patent LAs, and maximum LA diameter, were not significant predictors.

**Table 2:** Risk factors for aneurysm enlargement

Variables	Univariate	
	*p* Value	HR (95% CI)
Age	0.45	1.03 (0.95–1.13)
Male	0.026	0.23 (0.06–0.84)
Abdominal aorta		
Total volume	0.59	1.00 (0.99–1.01)
Mural thrombus volume ratio	0.003	0.88 (0.80–0.96)
Calcified volume ratio	0.50	0.94 (0.79–1.12)
Maximal aneurysm level		
Diameter	0.76	1.01 (0.93–1.11)
Patent LAs		
Number	0.94	0.98 (0.55–1.73)
Maximum diameter	0.67	1.48 (0.25–8.78)

HR: hazard ratio; CI: confidence interval; LA: lumbar artery

### ROC curve analysis

**Fig. 2:**
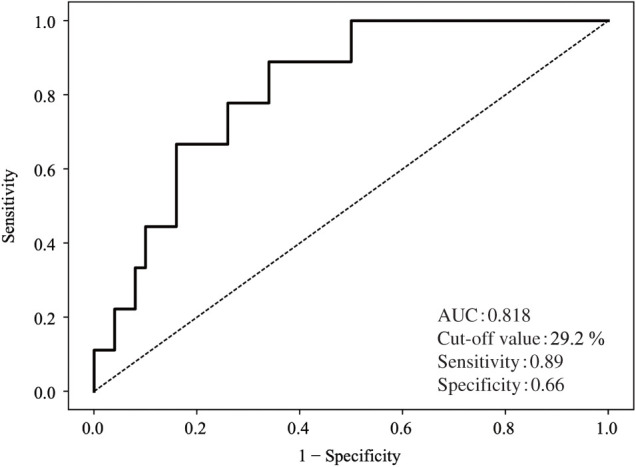
ROC curve for mural thrombus volume ratio. ROC curve analysis demonstrating the predictive performance of preoperative mural thrombus volume ratio for aneurysm enlargement in patients with lumbar artery–related type 2 endoleak. The AUC was 0.82, and the optimal cutoff value was 29.2%. ROC: receiver operating characteristic; AUC: area under the curve

The ROC curve analysis was performed to evaluate the predictive value of mural thrombus volume ratio for aneurysm enlargement (**Fig. 2**). The area under the ROC curve was 0.818, indicating good discriminative ability. The optimal cutoff value was identified as 29.2%, yielding a sensitivity of 88.9% and a specificity of 66.0%.

### Freedom from aneurysm enlargement

**Fig. 3:**
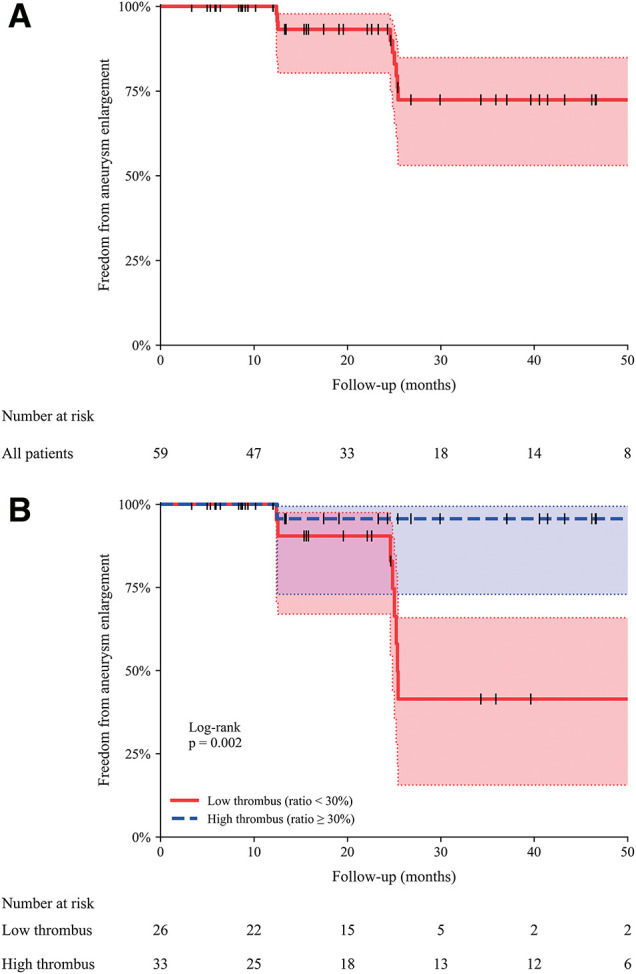
Freedom from aneurysm enlargement after endovascular aneurysm repair. (**A**) Kaplan–Meier curve illustrating freedom from aneurysm enlargement in the overall study population with lumbar artery–related type 2 endoleak after endovascular aneurysm repair. The estimated freedom from aneurysm enlargement at 50 months was 72.4% (95% confidence interval, 53.0%–84.8%). (**B**) Kaplan–Meier curves comparing freedom from aneurysm enlargement between patients stratified by preoperative mural thrombus volume ratio. Patients were classified into a low-thrombus group (<30%) and a high-thrombus group (≥30%) based on the optimal cutoff value derived from receiver operating characteristic analysis. Freedom from aneurysm enlargement was significantly lower in the low-thrombus group during follow-up (log-rank *p* = 0.002).

During follow-up, aneurysm enlargement occurred in 9 of 59 patients. **Figure 3A** shows the Kaplan–Meier estimate of freedom from aneurysm enlargement in the overall study population. The estimated freedom from aneurysm enlargement at 50 months was 72.4% (95% CI, 53.0–84.8). Kaplan–Meier analysis was subsequently performed after stratifying patients into 2 groups based on the mural thrombus volume ratio, using the optimal cutoff value of 29.2% derived from ROC curve analysis. Patients were categorized into a low-thrombus group (<30%) and a high-thrombus group (≥30%), and freedom from aneurysm enlargement was compared between the 2 groups (**Fig. 3B**).

Kaplan–Meier analysis demonstrated a significantly lower freedom from aneurysm enlargement in the low-thrombus group compared with the high-thrombus group. At 50 months, freedom from aneurysm enlargement was 41.5% in patients with a mural thrombus volume ratio <30% versus 95.7% in those with a ratio ≥30%, and this difference persisted throughout follow-up (log-rank *p* = 0.002).

## Discussion

T2EL after EVAR has been consistently associated with adverse long-term outcomes, including aneurysm enlargement, aneurysm-related mortality, rupture, and the need for secondary interventions.^1,2,5)^ A nationwide analysis by Seike et al. demonstrated that persistent T2EL is an independent risk factor for aneurysm enlargement and late adverse events.^1)^ Similarly, Sakaki et al. reported that approximately one-third of patients with T2EL developed aneurysm enlargement, with significantly higher aneurysm-related mortality compared with patients without T2EL.^2)^ Moreover, data from the ENGAGE registry indicated that up to 10% of patients with T2EL subsequently developed type 1 endoleak, suggesting that aneurysm enlargement associated with T2EL may contribute to progressive failure of proximal or distal sealing zones.^3)^

The incidence of T2EL has been reported to be relatively high in Japan, ranging from 28% to 46%, which is consistent with the 36.3% incidence observed in the present study.^1,2,4)^ Given this high prevalence, complete prevention of T2EL is unrealistic in routine clinical practice. In particular, routine embolization of all LAs is not feasible because of technical challenges, increased procedural complexity, and concerns regarding radiation exposure and contrast use.^9,10)^ Shirasu et al. demonstrated that radiation exposure increased substantially with embolization, from 424.6 Gy·cm^2^ in non-embolization procedures to 477.7 Gy·cm^2^ with IMA embolization alone, and further to 631.8 Gy·cm^2^ when LA embolization was added.^9)^ These findings highlight the potential patient-related and occupational risks associated with indiscriminate embolization strategies.

Instead, the clinically relevant issue is the identification of patients in whom T2EL leads to aneurysm enlargement, as this subgroup carries an increased risk of rupture and reintervention.^1,2,5)^ Therefore, appropriate risk stratification for aneurysm enlargement, rather than universal prevention of T2EL or indiscriminate embolization of LAs, should be the primary therapeutic focus.

Against this background, there is a clear need for a simple and reliable imaging-based risk stratification tool to identify patients with LA–related T2EL who are at high risk for aneurysm enlargement. Our findings indicate that the preoperative mural thrombus volume ratio fulfills this role, as it was significantly associated with aneurysm enlargement in univariable analysis and demonstrated good discriminative ability on ROC analysis. Accordingly, mural thrombus volume ratio may serve as a practical imaging biomarker to guide selective decision-making regarding preemptive embolization of LAs.^13,14)^

Several studies have examined predictors of aneurysm enlargement in patients with T2EL, primarily focusing on branch vessel anatomy, such as the number and diameter of patent aortic side branches, as well as hemodynamic parameters assessed by advanced imaging modalities, including 4-dimensional flow magnetic resonance imaging.^4,11,12,15)^ These studies have suggested that higher cumulative flow through branch vessels is associated with aneurysm enlargement. However, data specifically addressing T2EL caused exclusively by LAs are limited. Ueda et al. reported that, in patients with postoperative IMA occlusion, the number of persistent patent LAs was associated with aneurysm enlargement.^15)^ In contrast, the role of aneurysm wall–related factors, such as mural thrombus and calcification, has received comparatively little attention.^13,14,16)^

Previous investigations have suggested that a low mural thrombus burden is a predictor of the development and persistence of T2EL, presumably because a larger thrombus volume reduces both the number and flow capacity of patent branch vessels.^13,14)^ In the present study, which focused exclusively on patients with LA–related T2EL, mural thrombus volume ratio was identified as a significant predictor of aneurysm enlargement, whereas the number and maximum diameter of patent LAs were not. These findings indicate that mural thrombus is not merely a surrogate for branch vessel flow but may play a more direct biomechanical role in aneurysm behavior.

In addition to its biomechanical effects, arterial wall properties such as stiffness and thickness may also contribute to thrombus formation. Progressive stiffening and reduced compliance of the aneurysm wall can alter local hemodynamics, leading to disturbed flow patterns and increased flow turbulence near the wall, which may promote thrombus deposition and growth.^17,18)^ Once formed, mural thrombus has been shown to influence the biomechanical environment of the aneurysm wall. Experimental and clinical studies have demonstrated that mural thrombus can reduce wall stress within the aneurysm.^13)^ Speelman et al. showed that increased thrombus volume is associated with lower peak wall stress in abdominal aortic aneurysms.^19)^ The association between mural thrombus volume ratio and aneurysm enlargement should be interpreted cautiously. A low thrombus burden may reflect greater cumulative inflow from LAs, leading to persistent sac pressurization, rather than a direct causal biomechanical effect of mural thrombus itself. Thus, mural thrombus volume ratio may function as a surrogate imaging marker integrating both aneurysm wall characteristics and branch-vessel inflow dynamics. In addition, a significant sex difference was observed in the univariable analysis. Sex-related biological and clinical factors, including differences in aneurysm morphology, vessel wall properties, hormonal influences, and comorbidity profiles, may have influenced aneurysm behavior and contributed to the observed associations. However, given the limited number of aneurysm enlargement events, sex-stratified or adjusted analyses were not performed in the present study. In addition, the reproducibility of CT–based mural thrombus volumetry warrants consideration. Although a standardized, semi-automated analysis protocol was used in the present study, formal assessment of inter-observer and intra-observer variability was not performed. Therefore, further validation studies incorporating reproducibility analyses are required before widespread clinical application. These findings suggest that mural thrombus volume ratio may serve as a practical imaging marker for risk stratification when considering selective preemptive embolization of LAs. Consistent with this concept, our results suggest that patients with a preoperative mural thrombus volume ratio of ≥30% may experience a protective effect against aneurysm enlargement, even in the presence of persistent LA–related T2EL.

ROC curve analysis identified a mural thrombus volume ratio of 29.2% as the optimal cutoff for predicting aneurysm enlargement. For clinical applicability, patients were stratified using a threshold of 30%, which allowed clear risk discrimination. Kaplan–Meier analysis demonstrated a significantly lower freedom from aneurysm enlargement in patients with a mural thrombus volume ratio <30% during follow-up.

From a clinical perspective, our results support the selective use of preemptive embolization of LAs in patients with a mural thrombus volume ratio <30%. Because neither the number nor the diameter of patent LAs was associated with aneurysm enlargement, embolization strategies based solely on branch vessel anatomy may be insufficient. Instead, mural thrombus volume ratio may serve as a practical imaging biomarker to guide individualized decision-making regarding preemptive embolization, balancing potential benefits against procedural risks.^13,14)^

### Limitations

This study has several limitations. First, it was a retrospective, single-center analysis with a relatively small sample size and a limited number of aneurysm enlargement events (n = 9), which may restrict the generalizability of the findings. Because of the limited number of events, multivariable adjustment was not feasible, and residual confounding by measured and unmeasured variables cannot be excluded. Therefore, the association between mural thrombus volume ratio and aneurysm enlargement should be interpreted as exploratory and hypothesis-generating rather than independent or definitive.

Second, inter-observer and intra-observer reproducibility of mural thrombus and calcified volume measurements was not formally assessed. Although a standardized, semi-automated CT–based analysis protocol was applied and measurements were performed by a single blinded investigator, measurement variability cannot be entirely excluded. Further studies incorporating dedicated reproducibility testing are required to validate the robustness of this imaging biomarker.

Third, a significant sex difference was observed in univariable analysis; however, the limited number of aneurysm enlargement events precluded sex-stratified or sex-adjusted analyses. Therefore, residual confounding related to sex cannot be excluded. Larger prospective studies with standardized volumetric assessment and adequate event numbers are warranted to confirm and extend these findings.

## Conclusion

A low preoperative mural thrombus volume ratio was associated with aneurysm enlargement in patients with LA–related T2EL after EVAR. Given the technical challenges and potential risks associated with routine LA embolization, mural thrombus volume ratio may serve as a practical imaging biomarker to support selective decision-making regarding preemptive embolization in patients at higher risk of aneurysm enlargement.

## Supplementary Materials

Supplementary Fig. 1 Three-dimensional assessment of mural thrombus and calcification. (**A**) Three-dimensional reconstruction of the abdominal aorta obtained from contrast-enhanced CT, extending from just below the renal arteries to the aortic bifurcation. (**B**) Lumen segmentation of the abdominal aorta derived from contrast-enhanced CT images. (**C**) Three-dimensional visualization of mural thrombus volume, identified by subtracting the contrast-enhanced lumen volume from the total aortic volume obtained from non-contrast CT images. (**D**) Three-dimensional segmentation of calcified components within the aneurysm sac, identified on non-contrast CT using an Agatston threshold of >130 Hounsfield units.

## Declarations

### Data availability

Data cannot be shared publicly for ethical/privacy reasons. The data underlying this article cannot be shared publicly due to the privacy of individuals who participated in the study. On reasonable request, the data will be made available by the corresponding author after approval from the Medical Ethics Committee of Social Medical Corporation, Osaka International Medical & Science Center.
