# Supplementary figures and images for: Preoperative Mural Thrombus Volume Ratio Predicts Aneurysm Enlargement in Type 2 Endoleak after Endovascular Aortic Repair
Source: Ann Vasc Dis. 2026 Apr 1;19(1):26-00001. doi: 10.3400/avd.oa.26-00001 (PMC13051297; doi:10.3400/avd.oa.26-00001)

**Supplementary Fig. 1**

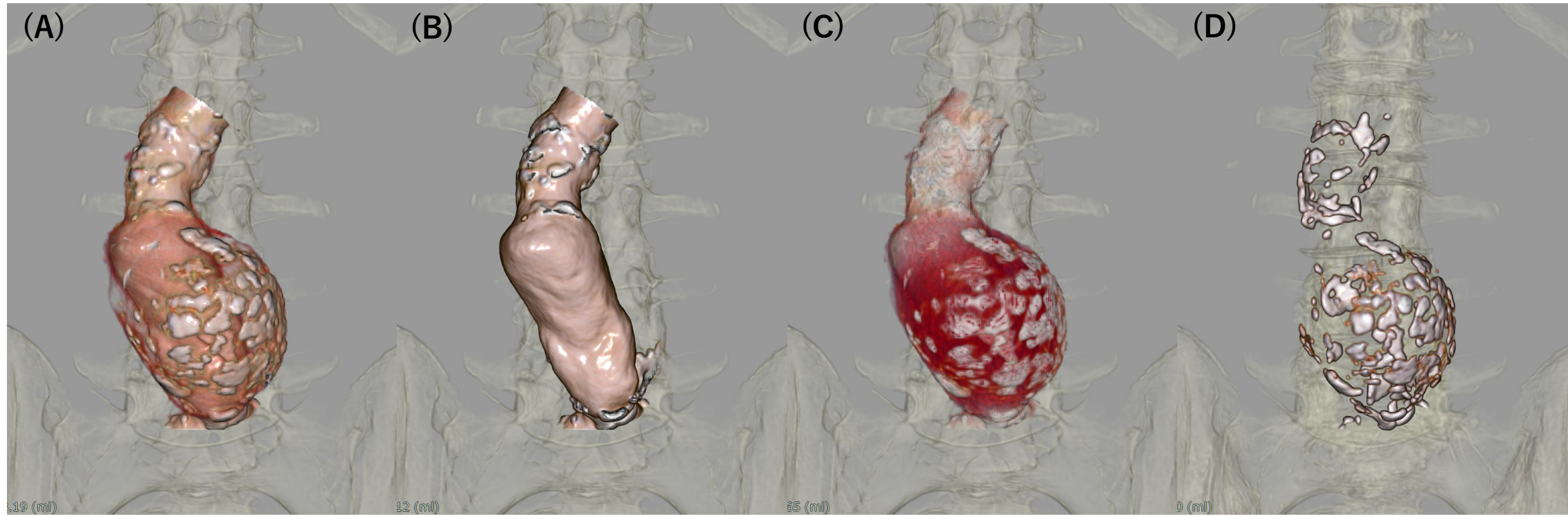

Supplement: Supplementary Fig. 1 — Three-dimensional assessment of mural thrombus and calcification. (A) Three-dimensional reconstruction of the abdominal aorta obtained from contrast-enhanced CT, extending from just below the renal arteries to the aortic bifurcation. (B) Lumen segmentation of the abdominal aorta derived from contrast-enhanced CT images. (C) Three-dimensional visualization of mural thrombus volume, identified by subtracting the contrast-enhanced lumen volume from the total aortic volume obtained from non-contrast CT images. (D) Three-dimensional segmentation of calcified components within the aneurysm sac, identified on non-contrast CT using an Agatston threshold of >130 Hounsfield units. [file avd-19-1-26-00001-s001.pdf]
